# Comparison of Three Nucleic Acid Amplification Tests and Culture for Detection of Group B *Streptococcus* from Enrichment Broth

**DOI:** 10.1128/JCM.01958-18

**Published:** 2019-05-24

**Authors:** Ji H. Shin, David T. Pride

**Affiliations:** a Department of Pathology, University of California, San Diego, La Jolla, California, USA; b Department of Medicine, University of California, San Diego, La Jolla, California, USA; Cleveland Clinic

**Keywords:** early-onset disease, GBS, group B *Streptococcus*, pregnancy, prenatal

## Abstract

Colonization of the gastrointestinal and genitourinary tracts of pregnant women with group B Streptococcus (GBS) can result in vertical transmission to neonates during labor/delivery. GBS infections in neonates can cause severe complications, such as sepsis, meningitis, and pneumonia. Accurate detection is critical because administration of intrapartum antibiotics can significantly reduce transmission. We compared the clinical sensitivities of three nucleic acid amplification tests (NAATs), the Hologic Panther Fusion GBS, Luminex Aries GBS, and Cepheid Xpert GBS LB assays, to that of the standard of care culture method recommended for GBS screening using 500 vaginal-rectal swab specimens after 18 to 24 h of broth enrichment. We identified 108 positive specimens (21.6%) by culture, while at least 1 of the 3 NAATs was positive for GBS in 155 specimens (31.0%). All 108 specimens positive by culture were also detected by the Panther Fusion assay, while 107/108 (99.1%) were detected by the Cepheid Xpert and Luminex Aries assays. Of the 61 specimens positive by at least 1 NAAT but negative by culture, 24 (39.3%) were positive by all 3 NAATs, suggesting that they represent true positives (TPs). NAATs offer less hands-on time, greater throughput, faster time to result, and potentially greater sensitivity than culture methods, and they should be considered the new gold standard for intrapartum GBS screening.

## Abstract (precis)

Colonization of the gastrointestinal and genitourinary tracts of pregnant women with group B Streptococcus (GBS) can result in vertical transmission to neonates during labor/delivery. GBS infections in neonates can cause severe complications, such as sepsis, meningitis, and pneumonia.

## INTRODUCTION

Streptococcus agalactiae, also known as group B *Streptococcus* (GBS), is a commensal Gram-positive bacterium that can transiently colonize the vagina, gastrointestinal tract, and urethra (1). While GBS usually results in asymptomatic colonization in healthy adults, it is more likely to cause invasive disease in immunocompromised individuals, newborns, and the elderly. Vaginal-rectal GBS colonization has been reported to occur in about 18% of pregnant women worldwide (with prevalence ranging from 13% to 35% depending on the region) (2) and in about 25% in the United States, according to the Centers for Disease Control and Prevention (CDC) (3). Spread of GBS to newborn children occurs via vertical transmission from vaginal-rectal-colonized pregnant women during labor and childbirth, and the organism may ascend the vagina to the amniotic fluid after the onset of labor and/or rupture of membranes. This transmission from the mother to the newborn occurs variably at an estimated rate of 40% to 73% (4), with about 1% to 2% of colonized newborns developing early-onset disease (EOD). EOD refers to symptoms of septicemia, meningitis, or pneumonia within the first 24 h to 7 days of delivery and may result in long-term disabilities, such as retardation, hearing or vision loss, and potentially death (5, 6). Worldwide, an estimated 205,000 infants acquired EOD, resulting in 90,000 deaths in infants of <3 months of age in 2015 and neurodevelopmental impairment in another 10,000 (7, 8). In the United States, GBS infection is the leading cause of infant morbidity and mortality and is the leading cause of bacterial meningitis and septicemia in a newborn’s first week of life (9).

The primary strategy for preventing EOD is intravenous intrapartum (during labor) antibiotic administration in women colonized with GBS. This strategy has been shown to be highly effective at reducing the incidence of vertical transmission (10). Thus, guidelines from the CDC recommend universal screening of antepartum women at 35 to 37 weeks of pregnancy to identify women colonized with GBS with the potential to transmit to their newborns during labor. These guidelines recommend using enriched culture-based methods using a vaginal-rectal specimen, followed by intrapartum antibiotic prophylaxis for women in whom GBS colonization has been identified (5, 10).

Systematic GBS screening efforts in the United States have resulted in a dramatic decrease in the incidence of EOD over the past 15 years, from 1.7 cases per 1,000 live births in the early 1990s to 0.34 to 0.37 cases in the early 2000s (10). Despite these rate decreases, EOD due to GBS remains a problem (11, 12). A large percentage (81%) of neonates who develop EOD are born from mothers with a negative GBS screening test, suggesting inadequate sensitivity of culture-based screening tests (false negatives [FNs]), or a change in the GBS colonization status of the mothers between screening and delivery (12, 13). CDC guidelines now include suggestions to improve the sensitivity of cultures, such as utilizing selective pigmented broths, DNA probes, or nucleic acid amplification tests (NAATs), but these are optional (10, 13).

Several Food and Drug Administration (FDA)-cleared real-time PCR-based NAATs are currently available for the qualitative detection of GBS in enriched broth cultures. NAATs on sample-to-answer platforms offer standardized processing technology for specimen extraction, amplification, and detection, provide a shorter turnaround time to result, and demonstrate improved GBS detection rates compared to those of culture (13–23).

Here, we evaluated the performances of three FDA-approved GBS NAATs, the Hologic Panther Fusion GBS assay, the Luminex Aries GBS assay, and the Cepheid Xpert GBS LB assay compared to that of the broth-enriched culture method recommended for GBS screening. All three assays were run on corresponding automated sample-to-answer systems. Using real-time PCR, the Aries and Xpert assays target a DNA region downstream of the CAMP factor (*cfb*) gene of GBS, and the Panther Fusion assay targets both the *cfb* genomic region and the *sip* (surface immunogenic protein) gene. We sought to determine the performance of each NAAT compared to that of conventional GBS culture, evaluate the analytical sensitivity of each test, and compare key metrics related to workflow.

## MATERIALS AND METHODS

### Specimen collection and enrichment.

Vaginal-rectal specimens (*n* = 500) were collected from pregnant women at 35 to 37 weeks of gestation at the University of California, San Diego, hospitals and clinics (between 26 February and 4 May 2018) using an ESwab Liquid Amies collection and transport system (Copan Diagnostics, Murrieta, CA). The women were from the following age groups: ≤25 years (*n *=* *61), 26 to 35 years (*n *=* *303), and 36 to 45 years (*n *=* *130). Specimens were directly inoculated within 24 h of collection into 5 ml of Lim enrichment broth (Todd Hewitt broth; Copan) with colistin and acid, and were incubated aerobically with 5% CO_2_ for 18 to 24 h at 35 to 37°C according to standard techniques.

### Sample processing.

After enrichment, an aliquot of the Lim broth was used for culture per the University of California, San Diego (UCSD) standard of care testing. Remaining enriched Lim broth specimens were aliquoted into multiple tubes for testing with each NAAT method. Samples were stored at 2 to 8°C for up to 72 h, and all tests were performed within 72 h of enrichment. The collection of the residual specimens used in this study was approved by the UCSD Institutional Review Board.

### Testing by culture.

Enriched Lim broths were immediately subcultured onto Trypticase soy agar plates containing 5% sheep blood for 48 h. Colonies suggestive of GBS were identified by the presence of beta hemolysis on blood agar plates (BAP). Presumptive GBS colonies were confirmed by matrix-assisted laser desorption ionization–time of flight (MALDI-TOF) mass spectrometry (Bruker Daltonics, Billerica, MA).

### Instruments.

The Panther Fusion GBS (Hologic, Inc., San Diego, CA), Aries GBS (Luminex Corp., Austin, TX), and Xpert GBS LB (Cepheid, Sunnyvale, CA) assays are all real-time PCR *in vitro* diagnostic tests approved for the qualitative detection of GBS from vaginal-rectal swabs from antepartum women following 18- to 24-h incubation in selective enrichment broth culture. All three assays are performed on sample-to-answer instruments (the Panther Fusion system, Aries system, and GeneXpert IV system, respectively), which automate DNA extraction, reagent preparation, nucleic acid amplification, and detection. Each assay includes an internal process control to account for amplification inhibition. At the time of this study, the Luminex Aries and Cepheid Xpert assays were FDA cleared and the Hologic Panther Fusion assay has since received FDA approval.

### Assay procedures.

Testing with the three NAATs was performed by a trained operator according to the manufacturers’ instructions. For the Panther Fusion assay, 1 ml of the enriched specimen was transferred to an Aptima specimen transfer tube containing 2.9 ml of specimen transport medium (STM). The cap was replaced and the sample was placed into a rack and loaded on the instrument, which has a capacity for 120 samples (8 racks with 15 samples each). Reagents for up to 336 GBS tests can be loaded onto the Panther Fusion system at any time. For the Aries assay, 200 μl of enriched specimen was transferred into the cassette sample chamber, which contained all assay components. The cassette sample chamber was capped, followed by the removal of the foil seal on the sample side of the cassette. Up to 6 cassettes can be placed into the Aries instrument with 2 cassette holders (accessible at UCSD) at a maximum capacity of 12 tests. For the Xpert assay, a single-use sterile disposable swab (part number SDPS-120; Cepheid) was dipped into the enriched specimen and placed directly into the sample chamber of the cartridge, which contained all assay components. The swab was snapped at the score mark, and the cartridge was loaded into the Cepheid GeneXpert IV system (available at UCSD), which has a capacity for 4 cartridges. Positive and negative controls were run on each day of testing; however, once verified by a clinical laboratory, controls were reduced in frequency according to the College of American Pathologists (CAP) guidelines for single-use assays with internal controls (once every 30 days or once when every new lot of cartridges is tested).

### Comparison to reference and consensus.

We used conventional culture as the primary reference method to determine the sensitivity and specificity of each NAAT assay. Because prior studies have indicated that NAAT testing generally is more sensitive than conventional cultures, we also compared the results of each assay to consensus results of each of the four assays performed. We then also determined the sensitivity and specificity of the NAATs compared to consensus results as a secondary method to provide some measure of how the NAATs compare with one another. This same method has been previously used to compare NAAT performance in GBS testing (13). The consensus result was determined for each sample, and samples were categorized as true positive (TP) if positive by two or more assays. Samples with invalid results were retested with the relevant assay, and the first valid result was used for test comparisons.

### Threshold cycle (*C_T_*).

Threshold cycle (*C_T_*) was determined for all NAAT-positive specimens to aid interpretation of the relative amounts of GBS nucleic acids present in specimens. *C_T_* is defined as the number of cycles required in a real-time PCR assay for the fluorescent signal to accumulate and cross the threshold (i.e., exceeds background level) to yield a positive result. *C_T_* levels are inversely proportional to the amount of target nucleic acid in the sample (i.e., the lower the *C_T_* level the greater the amount of target nucleic acid in the sample). In general, *C_T_* values of ≤30 indicate strong positive reactions indicative of abundant target nucleic acids in the sample. C*_T_* values of 31 to 36 indicate positive reactions indicative of moderate amounts of target nucleic acids. C*_T_* values of 37 to 40 indicate weak reactions indicative of minimal amounts of target nucleic acids, which could represent GBS colonization, environmental contamination, or false-positive (FP) results.

### Comparison of analytical sensitivity.

The analytical sensitivity of each NAAT assay was determined using strains of Streptococcus agalactiae serotypes III and V. Serial dilutions were made to represent 10, 30, 100, 300, 1,000, 3,000, 10,000 and 30,000 CFU per ml and tested in replicates of 10. Separate panels were made for each serotype. All panel members were prepared at the same time, aliquoted into individual tubes for each of ten replicates for each concentration, stored frozen, and thawed on the day of testing.

### Comparison of NAAT results.

To evaluate the performance between NAATs, the positive percent agreement and negative percent agreement were determined by comparing the result of each NAAT to the consensus (where at least 2/4 tests agree) result. The 95% confidence intervals (CI) for binomial proportions were calculated using the Wilson score method (24). To assess whether the performances of these tests were significantly different, a McNemar’s chi-square test was performed in GraphPad Prism. Discordant results were analyzed using the chi-square likelihood ratio test. A *P* value of <0.05 was considered significant.

## RESULTS

### Comparison of NAATs and culture results.

After enrichment, we tested 500 vaginal-rectal specimens from pregnant women by culture and the three NAATs to detect GBS. Each specimen was enriched in Lim broth for 18 to 24 h, and an aliquot was cultured on BAPs for 48 h. Three additional aliquots of the enriched broth were stored at 2 to 8°C, and each tested on the three NAAT platforms within 72 h. A total of 19 (3.8%) Aries samples produced initial invalid results, but produced valid results (4 positive and 15 negative) upon repeat testing. No invalid results were obtained with the Panther Fusion or Xpert assays. Culture was positive for 108 (21.6%) specimens, and initial positives for NAATs were 143 (28.6%) for the Panther Fusion, 147 (29.4%) for the Xpert, and 155 (31.0%) for Aries assays (Table 1). All 108 culture-positive specimens were also positive by all three NAATs, apart from one specimen which was positive by culture and by the Panther Fusion assay but which tested negative by the other NAATs.

**TABLE 1:** Comparison of NAATs to culture results

Assay	Assay result	Culture result		Total
		Positive	Negative	
Panther Fusion	Positive	108	35	143
	Negative	0	357	357
Aries	Positive	107	48	155
	Negative	1	344	345
Xpert	Positive	107	40	147
	Negative	1	352	353
All assays		108	392	500

### Method comparison to consensus result.

We defined the consensus as 2 or more tests that produced concordant results and used the results to define true positives and true negatives (TNs). Using this criterion, 147 specimens (29.4%) were defined as true positives and 353 were defined as true negatives. The rate of GBS detection was 21.6% (108/500) with culture, 28.2% (141/500) for the Panther Fusion and Xpert assays, and 28.4% (142/500) for the Aries assay (Table 2). Compared to the consensus result, culture produced 39 putative false-negative results, while the Panther Fusion, Aries, and Xpert assays produced 6, 5, and 6, respectively. No putative false-positive results were observed with culture, while 2, 13, and 6 were recorded for the Panther Fusion, Aries, and Xpert assays, respectively (Table 2).

**TABLE 2:** Performance of NAATs compared to consensus results for detection of GBS

Method	No. of results^*a*^				Sensitivity (95% CI)	Specificity (95% CI)
	TP	TN	FP	FN		
Culture	108	353	0	39	73.5% (65.8–80.0)	100.0% (98.9–100)
Panther Fusion	141	351	2	6	95.9% (91.4–98.1)	99.4% (98.0–99.8)
Aries	142	340	13	5	96.6% (92.3–98.6)	96.3% (93.8–97.8)
Xpert	141	347	6	6	95.9% (91.4–98.1)	98.3% (96.3–99.2)

a TP, true positive; TN, true negative; FP, false positive; FN, false negative; CI, confidence interval.

We calculated putative sensitivity and specificity for each assay based on consensus results. The putative sensitivities of the Panther Fusion assay and the Xpert GBS assay were identical at 95.9% (95% CI, 91.4 to 98.1%). The putative sensitivity for the Aries GBS assay was 96.6% (95% CI, 92.3 to 98.6). Specificities for each assay were 99.4% (95% CI, 98.0 to 99.8%), 98.3% (95% CI, 96.3 to 99.2%), and 96.3% (95% CI, 93.8 to 97.8%) for the Panther Fusion, Xpert, and Aries assays, respectively (Table 2). No significant differences were identified in putative sensitivity or specificity between the three NAATs and the consensus result (McNemar’s chi-square test, *P > *0.05). In contrast, all three NAATs were significantly more sensitive than the culture method when using the consensus method (*P < *0.0001). Percent agreement between the 3 NAATs was as follows: 94.8% concordance between Panther Fusion and Aries (κ = 0.88 [95% CI, 0.83 to 0.92]), 96.0% concordance between Panther Fusion and Xpert (κ = 0.90 [95% CI, 0.86 to 0.95]), and 94.4% concordance between Aries and Xpert (κ = 0.87 [95% CI, 0.82 to 0.92]).

### Threshold cycle (*C_T_*).

**FIG 1:**
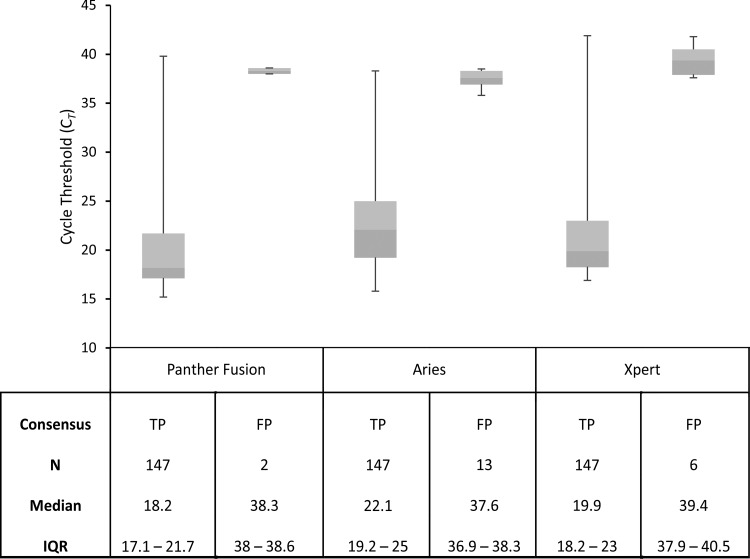
Box plot diagram (top) and table (bottom) depicting the *C_T_* values for GBS detection by NAATs. The bottom, middle, and top lines of each box correspond to the 25%, 50%, and 75% cumulative frequencies of the observed values, respectively. The endpoints of the whiskers show the 2.5th and 97.5th percentiles. The number of specimens (N), median *C_T_* values, and interquartile ranges (IQR) are depicted in the bottom table.

To facilitate interpretation of discordant results between the NAATs, *C_T_* values for positive NAAT results were analyzed to measure distribution and interquartile ranges (IQRs) of *C_T_* values for putative true-positive (TP) and false-positive (FP) GBS specimens (Fig. 1). For TPs (*n *=* *147), the median *C_T_* values were 18.2 (IQR, 17.1 to 21.7), 22.1 (IQR, 19.2 to 25), and 19.9 (IQR, 18.2 to 23) for the Panther Fusion, Aries, and Xpert assays, respectively. The mean *C_T_* values plus or minus standard deviation (SD) were 22.0 ± 7.5, 24.0 ± 6.2, and 23.2 ± 7.0, respectively. For FPs (*n *=* *21), the median *C_T_* value was 38.3 (IQR, 38 to 38.6, *n *=* *2), 37.6 (IQR, 36.9 to 38.3, *n *=* *13), and 39.4 (IQR, 37.9 to 40.5, *n *=* *6), and the mean *C_T_* ± SD was 38.3 ± 0.4, 37.5 ± 0.8, and 39.4 ± 1.5 for each NAAT test, respectively.

We also determined the mean NAAT *C_T_* value after samples were categorized according to the rate of agreement among the 4 methods (Table 3). For samples with 100% positive agreement among all 4 methods (positive by culture and all 3 NAATs, *n *=* *107), the NAAT *C_T_* values ranged between 18.6 and 21.4 (Table 3), indicating high quantities of GBS DNA. The average NAAT *C_T_* value for samples with 100% positive agreement between the 3 NAATs (negative by culture and positive by all three NAATs, *n *=* *24) was higher (ranging from 30.2 to 30.8) but nevertheless suggests a high quantity of target DNA in these specimens. Samples positive by two NAATs (*n *=* *15) had an average NAAT *C_T_* value ranging from 36.2 to 39.0, representing a low to moderate amount of target in many of those samples. The average NAAT *C_T_* values for samples with the lowest method concordance rate (positive by a single NAAT, *n *=* *21) ranged from 37.5 to 39.4, demonstrating the lowest target content observed for the putative FPs.

**TABLE 3:** Average *C_T_* value by method concordance rate

Assay	Average *C_T_* value for^*a*^:			
	4 methods	3 NAATs	2 NAATs	1 NAAT
Panther Fusion	18.6 ± 2.6	30.8 ± 8.1	37.2 ± 4.6	38.3 ± 0.4
Aries	21.4 ± 3.3	30.2 ± 6.5	36.2 ± 1.9	37.5 ± 0.8
Xpert	20.0 ± 2.5	30.8 ± 7.4	39.0 ± 1.1	39.4 ± 1.5

a *C_T_*, threshold cycle; NAAT, nucleic acid amplification test.

### Comparison of analytical performance.

We evaluated the analytical sensitivity of each assay to decipher if there may be differences in the ability of each assay to detect GBS in contrived specimens. We contrived specimens with GBS serotypes III and V at 8 separate final concentrations (10, 30, 100, 300, 1,000, 3,000, 10,000 and 30,000 CFU/ml) in Lim broth and created 10 replicates of each. We chose serotypes III and V because we wanted to test analytical sensitivity across multiple serotypes, and these serotypes represent two of the most commonly identified GBS serotypes in recto-vaginal specimens (25). The Panther Fusion assay detected 10/10 replicates at 300 CFU/ml and 2/10 replicates at 30 CFU/ml for both serotypes (Table 4). The Cepheid assay detected 10/10 at 3,000 CFU/ml for both serotypes, 2/10 at 100 CFU/ml for serotype III, and 0/10 at 100 CFU/ml for serotype V. The Aries assay detected 10/10 at 30,000 CFU/ml for both serotypes but only detected 8/10 for both serotypes at 10,000 CFU/ml.

**TABLE 4:** Comparison of analytical performance

CFU/ml	No. of isolates identified by serotype and assay (*n*/total no. of isolates)					
	Serotype III			Serotype V		
	Panther Fusion	Cepheid	Aries	Panther Fusion	Cepheid	Aries
30,000	10/10	10/10	10/10	10/10	10/10	10/10
10,000	10/10	10/10	8/10	10/10	10/10	8/10
3,000	10/10	10/10	0/10	10/10	10/10	0/10
1,000	10/10	9/10	0/10	10/10	9/10	0/10
300	10/10	2/10	0/10	10/10	5/10	0/10
100	8/10	2/10	0/10	7/10	0/10	0/10
30	2/10	0/10	0/10	2/9	0/10	0/10
10	0/10	0/10	0/10	1/10	0/10	0/10
0	0/10	0/10	0/10	0/10	0/10	0/10

### Workflow of automated platforms.

In addition to assay performance and instrument reagent costs, workflow metrics, such as time to result, test capacity, and throughput, should be considered because they can influence efficiency and labor costs (26). We compared these metrics for each platform, excluding any assay preparation times. Results from testing a single specimen on the Panther Fusion system were available in 2 h and 24 min, followed by the release of 5 results every 5 min (i.e., 1 sample per minute), yielding a throughput of 1,005 samples in a 24-h period (Table 5). Results for up to 12 specimens on the Aries system were available in 2 h, providing a throughput of 144 specimens per instrument in 24 h. The Xpert IV system in our laboratory required 1 h to report up to 4 results, processing 96 samples in 24 h. The capacity for specimen loading per instrument was 120 for Panther Fusion, 12 for Aries, and 4 for Xpert. While the GeneXpert IV and Aries were the instrument versions accessible for this study, other instruments, such as the GeneXpert Infinity 80 (up to 80 specimens at a time), could produce up to 1,920 specimens in 24 h, while additional Aries machines would lead to greater throughput as well.

**TABLE 5:** Description of automated platforms

Instrument	Manufacturer	Configuration	Sample capacity (no.)^*a*^	TTFR (h)^*b*^	Throughput in 24 h^*c*^
Panther Fusion	Hologic	Nonbatch, random-access system; separate consumables	120	2.4	1,005
GeneXpert IV	Cepheid	Nonbatch, random-access system	4	1	96
GeneXpert Infinity 80	Cepheid	Nonbatch, random-access system	80	1	1,920
Aries M1	Luminex	Nonbatch, random-access system	6	2	72
Aries	Luminex	Nonbatch, random-access system	12	2	144

a Maximum number of samples processed without a return visit. Sample can be a specimen or a control.

b TTFR, time to first result, the time from sample loading to availability of result. Does not include sample preparation times.

c Maximum number of specimens processed in 24 h with return visits, per instrument.

## DISCUSSION

Since the CDC guidelines issued in 2002, culture has been the gold standard for the screening of GBS colonization in prepartum women. Even though culture-based GBS screening has dramatically reduced the incidence of EOD and its associated morbidity and mortality, culture in this and other studies has a sensitivity of 53 to 70% (13, 22), which fails to meet the 90% sensitivity threshold recommended by current CDC guidelines (10). NAATs offer the potential to meet this sensitivity threshold, but do have limitations that must also be considered. For example, antibiotic susceptibility testing, which is particularly important in individuals with GBS colonization who also have beta lactam allergies (27) is generally not available for NAATs. Also, FDA-approved NAAT tests for GBS detection continue to require broth enrichment, with the exception of the Xpert GBS assay from Cepheid, which is approved to use during labor and delivery but not for screening prior to labor and delivery (28).

In the present study, we compared the recently FDA-cleared Panther Fusion GBS assay, which uses real-time PCR, with two other FDA-cleared real-time PCR NAATs (Aries and Xpert) and standard culture for the detection of GBS in vaginal-rectal specimens in pregnant women. Our main finding was that that each of the three NAATs was able to detect most of the culture-positive specimens; only the Panther Fusion assay detected all culture-positive specimens, while the Xpert and Aries assays each detected all but one culture-positive specimen. There were 24 specimens negative by culture but positive by all 3 NAATs. These specimens had mean *C_T_* values of 30.2 to 30.8, suggesting that there were high levels of nucleic acids in each. These results also suggest that all three NAATs had significantly higher sensitivity than culture (by ∼25%; *P* < 0.0001), which is consistent with prior published results (13, 22) and reinforces the limitations of culture-based screening for GBS detection. In our analysis of the consensus results, culture likely missed over a quarter (39/147) of GBS-positive samples, yielding a putative sensitivity of 73.5%, while all three NAATs had a putative sensitivity above 95%. These findings are consistent with those of two publications that compared commercially available NAATs with culture. In the study by Couturier et al., which compared the performance of 3 NAATs (BD Max GBS, Illumigene GBS, and Quidel AmpliVue GBS) with culture, NAAT sensitivity ranged from 98.5% to 100%, while culture sensitivity was 70.6% (22). A similar study comparing the same three NAATs with culture found that NAATs were from 37.3% to 46.4% more sensitive than culture for GBS detection (NAATs, 90.9% to 100%; culture, 53.6%) (13). In both of these studies, the agreement among the NAATs was high, 97.1% to 98.4% in Couturier’s study (22) and 92% to 93% in Miller’s study (13). In our study, the agreement among Panther Fusion, Aries and Xpert GBS assays was >97%. Another study found a 97% agreement between four NAATs (BD Max, Illumigene, Aries, and Xpert) (14). The results of this and other studies highlight the underestimation of GBS colonization with broth-enriched culture methods and support the adoption of NAATs as the new gold standard to improve GBS detection.

We attempted to evaluate the relative analytical sensitivity of the GBS NAATs, and identified substantial differences between each of the assays using 2 GBS serotypes. Our study suggests the Panther Fusion assay is more sensitive than the other assays despite there being little difference in the clinical sensitivity. While the results suggested the Panther Fusion assay had the greatest analytical sensitivity, we observed the greatest numbers of positive clinical specimens in the Aries assay, followed by the Xpert assay. By our consensus criterion, there were 2 FPs for the Panther fusion, 6 for the Xpert, and 13 for the Aries. The potential FPs on each of these assays correlates to relatively high *C_T_* values associated with these specimens, which often correlate with false-positive results (22). These results, however, could also represent low-level positives that could not be detected by all assays. FP results can lead to unnecessary antibiotic treatment which can have harmful effects, such as severe maternal allergic reactions, increase in drug-resistant organisms, exposure of newborn infants to resistant bacteria, and postnatal maternal and neonatal yeast infections (1).

We noted the high rate of initial invalid results for Aries compared to the other platforms, which produced no invalid results during the course of this study. Aries had 19/500 (3.8%) initial invalid results, which all yielded valid results upon repeat testing. We initially believed the invalid results may be related to workflow or familiarity of the staff with the assay; however, the invalid results occurred throughout the course of the study, suggesting that familiarity with the platform did not affect the invalid rate. Both the Aries and the Xpert systems were used for other routine clinical testing before and during this study, so familiarity of the staff with these platforms is unlikely to account for Aries’ high invalid rate. In addition, another report also produced a high rate of invalid results (2%; 14/688) with the GBS test on the Aries (15). An alternative explanation could be that the Aries GBS assay is more susceptible to specimen collection practices such as the amount of lubricant used or mucous obtained during specimen collection (16). We were not able to investigate this possibility further because the specimens had already been deidentified prior to testing.

It is now widely believed that NAATs can significantly improve sensitivity for GBS detection over conventional culture methods (18), as we have also demonstrated in this study. We compared the NAAT platforms with subculture, using 5% sheep’s blood agar plates to identify beta-hemolytic bacteria and then verifying that those bacteria are GBS. The major limitation of this method is that approximately 5% to 8% of GBS are not hemolytic because they lack a beta-hemolysin (29–31), and thus they cannot be identified using the testing protocol we used. Thus, some potential culture positives could have gone unrecognized in this study because of the reliance on beta hemolysis. There are other methods, such as subculture to Granada agar (31), which can identify beta-hemolytic GBS, but they generally suffer from the same sensitivity problem as blood agar when GBS strains lack a beta-hemolysin (30). Carrot broth is based on Granada medium (32), and in some studies it has been shown to have greater sensitivity for GBS detection (33). Most cultivation-based GBS detection techniques suffer from low sensitivity when GBS relative abundances are low (34). The data in this study are consistent with other studies that did use cultivation methods that include methods to identify nonhemolytic GBS strains; thus, the lack of methods to identify these nonhemolytic strains likely does not impact the main findings of this study. While NAATs have some clear advantages over conventional cultures, in individuals with beta-lactam allergies for whom antibiotic susceptibilities for GBS are needed, laboratories using NAAT testing only cannot obtain antibiotic susceptibilities. Furthermore, as our data indicate, in individuals who are NAAT positive, there may be difficulty in recovering the GBS to perform antibiotic susceptibility tests (19).

### Conclusions.

Compared to standard culture, the use of commercially available real-time PCR NAATs for GBS screening in pregnant women has the potential to increase the overall sensitivity of GBS screening (by reducing the number of false negatives) while reducing the time to obtaining results. By improving GBS detection, NAAT-based screening has the potential to significantly reduce the neonate morbidity and mortality associated with EOD. The findings in this study support the use of highly sensitive real-time PCR NAATs as the preferred method for GBS screening in the prenatal period. Our results indicate that each of the three tests utilized has the potential to significantly increase sensitivity for GBS detection and reduce turnaround times while holding the potential to reduce infant morbidity/mortality particularly in large medical centers. Further studies to address the clinical impact of NAAT platforms compared to that of conventional cultures are necessary to elucidate their potential benefits in reducing EOD and potentially reducing infant/neonate mortality.
